# Supplementary material for: Body size as a metric for the affordable world
Source: eLife. 2024 Mar 28;12:RP90583. doi: 10.7554/eLife.90583 (PMC10987089; doi:10.7554/eLife.90583)
Supplement: Supplementary file 1. [file elife-90583-supp1.docx]

Supplementary file 1a. Cortical regions showing object-selective activation for whole-brain conjunction analysis (R = right hemisphere, L = left hemisphere; Z > 2.3, *p* = 0.05, cluster corrected)

| **Cluster** | **Region** | **Number of voxels in region** | **MNI coordinates** | | | **Peak Z value** |
| --- | --- | --- | --- | --- | --- | --- |
|  |  |  | **x** | **y** | **z** |  |
| 1 | L Postcentral Gyrus | 1804 | -54 | -26 | 50 | 5.41 |
|  | L Middle Occipital Gyrus | 1392 |  |  |  |  |
|  | L Inferior Parietal Lobule | 1321 |  |  |  |  |
|  | L Superior Parietal Lobule | 1284 |  |  |  |  |
|  | L Supplementary Motor Area | 851 |  |  |  |  |
|  | L Precentral Gyrus | 749 |  |  |  |  |
|  | L Inferior Occipital Gyrus | 409 |  |  |  |  |
|  | L Middle Temporal Gyrus | 402 |  |  |  |  |
|  | L Fusiform Gyrus | 390 |  |  |  |  |
|  | L Precuneus | 320 |  |  |  |  |
|  | L Inferior Temporal Gyrus | 303 |  |  |  |  |
| 2 | R Middle Occipital Gyrus | 594 | 28 | -90 | 12 | 4.55 |
|  | R Middle Temporal Gyrus | 335 |  |  |  |  |
|  | R Fusiform Gyrus | 321 |  |  |  |  |
|  | R Inferior Temporal Gyrus | 209 |  |  |  |  |
|  | R Inferior Occipital Gyrus | 145 |  |  |  |  |
| 3 | R Postcentral Gyrus | 896 | 30 | -58 | 58 | 3.90 |
|  | R Precentral Gyrus | 577 |  |  |  |  |
|  | R Superior Parietal Lobule | 427 |  |  |  |  |
| 4 | L Rolandic Operculum | 331 | -50 | 8 | 4 | 3.97 |
|  | L Inferior Frontal Gyrus, opercular part | 268 |  |  |  |  |
| 5 | L Middle Frontal Gyrus | 520 | -28 | 46 | 8 | 3.68 |
|  | L Inferior Frontal Gyrus, triangular part | 245 |  |  |  |  |

Supplementary file 1b. Cortical regions reaching significance in the contrasts of (A) objects within versus objects beyond and (B) objects beyond versus objects within, whole-brain analysis (R = right hemisphere, L = left hemisphere; Z > 2.3, *p* = 0.05, cluster corrected)

| **Cluster** | **Region** | **Number of voxels in region** | **MNI coordinates** | | | **Peak Z value** |
| --- | --- | --- | --- | --- | --- | --- |
|  |  |  | **x** | **y** | **z** |  |
| Objects within > Objects beyond | | | | | | |
| 1 | L Cuneus | 363 | 10 | -90 | 20 | 4.24 |
|  | R Cuneus | 309 |  |  |  |  |
|  | L Lingual Gyrus | 176 |  |  |  |  |
|  | R Lingual Gyrus | 293 |  |  |  |  |
| Objects beyond > Objects within | | | | | | |
| 1 | L Middle Occipital Gyrus | 1831 | -16 | -92 | -8 | 5.72 |
|  | L Fusiform Gyrus | 994 |  |  |  |  |
|  | L Superior Parietal Lobule | 584 |  |  |  |  |
|  | L Lingual Gyrus | 504 |  |  |  |  |
|  | L Inferior Occipital Gyrus | 304 |  |  |  |  |
|  | L Superior Occipital Gyrus | 211 |  |  |  |  |
|  | L Parahippocampal Gyrus | 210 |  |  |  |  |
|  | L Precuneus | 205 |  |  |  |  |
| 2 | R Middle Occipital Gyrus | 1358 | 20 | -86 | -2 | 5.40 |
|  | R Lingual Gyrus | 340 |  |  |  |  |
|  | R Superior Parietal Lobule | 328 |  |  |  |  |
|  | R Superior Occipital Gyrus | 320 |  |  |  |  |
|  | R Inferior Occipital Gyrus | 276 |  |  |  |  |
| 3 | R Fusiform Gyrus | 483 | 34 | -38 | -16 | 4.73 |
|  | R Parahippocampal Gyrus | 316 |  |  |  |  |

Supplementary file 1c. Cortical regions reaching significance in contrasts of (A) congruent versus incongruent and (B) incongruent versus congruent, whole-brain analysis (R = right hemisphere, L = left hemisphere; Z > 2.3, *p* = 0.05, cluster corrected)

| **Cluster** | **Region** | **Number of voxels in region** | **MNI coordinates** | | | **Peak Z value** |
| --- | --- | --- | --- | --- | --- | --- |
|  |  |  | **x** | **y** | **z** |  |
| Congruent > Incongruent | | | | | | |
| 1 | L Inferior Parietal Lobule | 322 | -44 | -50 | 64 | 3.47 |
| 2 | R Superior Parietal Lobule | 339 | 36 | -66 | 52 | 3.31 |
|  | R Inferior Parietal Lobule | 165 |  |  |  |  |
| Incongruent > Congruent | | | | | | |
| - | *No significant cluster* | - | - | - | - | - |

Supplementary file 1d. Cortical regions showing significant interaction between object type and congruency, whole-brain analysis (OW = Objects within, OB = Objects beyond; R = right hemisphere, L = left hemisphere; Z > 2.3, *p* = 0.05, cluster corrected)

| **Cluster** | **Region** | **Number of voxels in region** | **MNI coordinates** | | | **Peak Z value** |
| --- | --- | --- | --- | --- | --- | --- |
|  |  |  | **x** | **y** | **z** |  |
| (OW_Congruent – OW_Incongruent ) > (OB_Congruent – OB_Incongruent ) | | | | | | |
| 1 | L Middle Occipital Gyrus | 831 | 22 | -94 | 10 | 4.25 |
|  | R Middle Occipital Gyrus | 187 |  |  |  |  |
|  | L Fusiform Gyrus | 113 |  |  |  |  |
|  | R Fusiform Gyrus | 376 |  |  |  |  |
|  | L Inferior Occipital Gyrus | 293 |  |  |  |  |
|  | R Inferior Occipital Gyrus | 276 |  |  |  |  |
|  | L Lingual Gyrus | 215 |  |  |  |  |
|  | R Lingual Gyrus | 345 |  |  |  |  |
|  | L Superior Occipital Gyrus | 123 |  |  |  |  |
| 2 | R Supplementary Motor Area | 383 | 14 | 14 | 60 | 3.39 |
| 3 | R Superior Parietal Lobule | 191 | 36 | -62 | 56 | 3.18 |
|  | R Inferior Parietal Lobule | 114 |  |  |  |  |
| 4 | R Insula | 175 | 32 | 18 | 8 | 3.41 |
| (OB_Congruent – OB_Incongruent ) > (OW_Congruent – OW_Incongruent ) | | | | | | |
| - | *No significant cluster* | - | - | - | - | - |

Supplementary file 1e. Cortical regions showing significant congruency effect (congruent versus incongruent) for objects within, whole-brain analysis (R = right hemisphere, L = left hemisphere; Z > 2.3, *p* = 0.05, cluster corrected)

| **Cluster** | **Region** | **Number of voxels in region** | **MNI coordinates** | | | **Peak Z value** |
| --- | --- | --- | --- | --- | --- | --- |
|  |  |  | **x** | **y** | **z** |  |
| 1 | L Middle Occipital Gyrus | 820 | -14 | -96 | -2 | 4.29 |
|  | R Middle Occipital Gyrus | 214 |  |  |  |  |
|  | L Inferior Occipital Gyrus | 367 |  |  |  |  |
|  | R Inferior Occipital Gyrus | 487 |  |  |  |  |
|  | L Lingual Gyrus | 329 |  |  |  |  |
|  | R Lingual Gyrus | 484 |  |  |  |  |
|  | L Fusiform Gyrus | 341 |  |  |  |  |
|  | R Fusiform Gyrus | 463 |  |  |  |  |
|  | R Inferior Temporal Gyrus | 280 |  |  |  |  |
| 2 | R Superior Parietal Lobule | 891 | 32 | -58 | 50 | 3.80 |
|  | R Inferior Parietal Lobule | 619 |  |  |  |  |
|  | R Supramarginal Gyrus | 300 |  |  |  |  |
|  | R Precuneus | 294 |  |  |  |  |
|  | L Superior Parietal Lobule | 210 |  |  |  |  |
| 3 | L Inferior Parietal Lobule | 668 | -48 | -46 | 60 | 3.82 |
| 4 | R Superior Frontal Gyrus | 501 | 16 | 12 | 60 | 3.57 |
|  | R Supplementary Motor Area | 200 |  |  |  |  |
| 5 | R Middle Frontal Gyrus | 398 | 40 | 42 | 34 | 3.44 |
| 6 | R Inferior Frontal Gyrus, opercular part | 384 | 50 | 14 | 38 | 3.33 |
