# Supplementary material for: Body size as a metric for the affordable world
Source: eLife. 2024 Mar 28;12:RP90583. doi: 10.7554/eLife.90583 (PMC10987089; doi:10.7554/eLife.90583)
Supplement: Supplementary file 2. [file elife-90583-supp2.docx]

Supplementary file 2. The full list of inanimate objects used in the behavioral study, with the corresponding size rank noted according to Konkle and Oliva (2011).

| **Object** | **Diagonal Size (cm)** | **Size Rank** |
| --- | --- | --- |
| airplane | 7,618 | 8 |
| apple | 14 | 2 |
| ball | 40 | 3 |
| bed | 252 | 6 |
| bike | 181 | 5 |
| bottle | 35 | 3 |
| brick | 25 | 3 |
| chair | 115 | 5 |
| eggplant | 31 | 3 |
| hammer | 25 | 3 |
| kettle | 30 | 3 |
| ladder | 213 | 6 |
| laptop | 44 | 4 |
| phone | 17 | 2 |
| piano | 184 | 6 |
| pipa | 107 | 4 |
| plate | 20 | 2 |
| potted plant | 24 | 3 |
| shoe | 32 | 3 |
| skateboard | 83 | 4 |
| sweater | 99 | 4 |
| tent | 500 | 7 |
| tree | 3,016 | 8 |
| umbrella | 104 | 4 |
